# 354. SARS-CoV-2 Viral Viability Culture and Sequencing from Immunocompromised Patients with Persistently Positive SARS-CoV-2 PCR Results

**DOI:** 10.1093/ofid/ofab466.555

**Published:** 2021-12-04

**Authors:** Abby Sung, Adam Bailey, Meghan Wallace, Henry B Stewart, David McDonald, Candace R Miller, Kimberly Reske, Caroline O’Neil, Victoria J Fraser, Victoria J Fraser, Michael S Diamond, Carey-Ann Burnham, Carey-Ann Burnham, Hilary Babcock, Hilary Babcock, Jennie H Kwon

**Affiliations:** 1 Washington University School of Medicine in St. Louis, Saint Louis, Missouri; 2 UW-Madison, Madison, Wisconsin; 3 Washington University, St. Louis, Missouri; 4 Washington University in St. Louis, St. Louis, MO; 5 Washington University School of Medicine, St. Louis, MO

## Abstract

**Background:**

Immunocompromised (IC) patients (pts) can have prolonged SARS-CoV-2 PCR positivity, even after resolution of COVID-19 symptoms. This study aimed to determine if viable virus could be detected in samples collected > 21 days after an initial positive (pos) SARS-CoV-2 PCR in IC pts.

**Methods:**

We obtained 20 remnant SARS-CoV-2 PCR pos nasopharyngeal swabs from IC pts (bone marrow or solid organ transplant, high dose steroids, immunosuppressive medications) with a pos repeat PCR within the previous 30 days. The repeat specimens were cultured on Vero-hACE2-TMPRSS2 cells and incubated for 96 hours to assess viral viability. Viable RNA and infectious virus in the cultured cells were measured by qPCR and infectious plaque assays. RNA sequencing was performed on a HiSeq platform (Illumina). Samples also underwent SARS-CoV-2 antigen (Ag) testing (BD Veritor). Clinical data were extracted from the electronic health record by chart review.

**Results:**

Pt characteristics are in Table 1. Viral cultures from the repeat specimen were negative (neg) for 18 pts and pos for 2 (Table 2). Pt 1 is a 60M treated with obinatuzumab 19 days prior to his first pos PCR test, with repeat specimen collected 21 days later (cycle threshold (Ct) not available). Pt 1 had a low viral titer (27 PFU/mL) & a D614G mutation on sequencing. Pt 2 is a 75M treated with rituximab 10 days prior to his first pos PCR test, with repeat specimen collected 23 days later (Ct 27.56/27.74). Pt 2 had a high viral titer (2e6 PFU/mL) and D614G, S98F, and S813I mutations.

Demographics of Study Population (N=20)

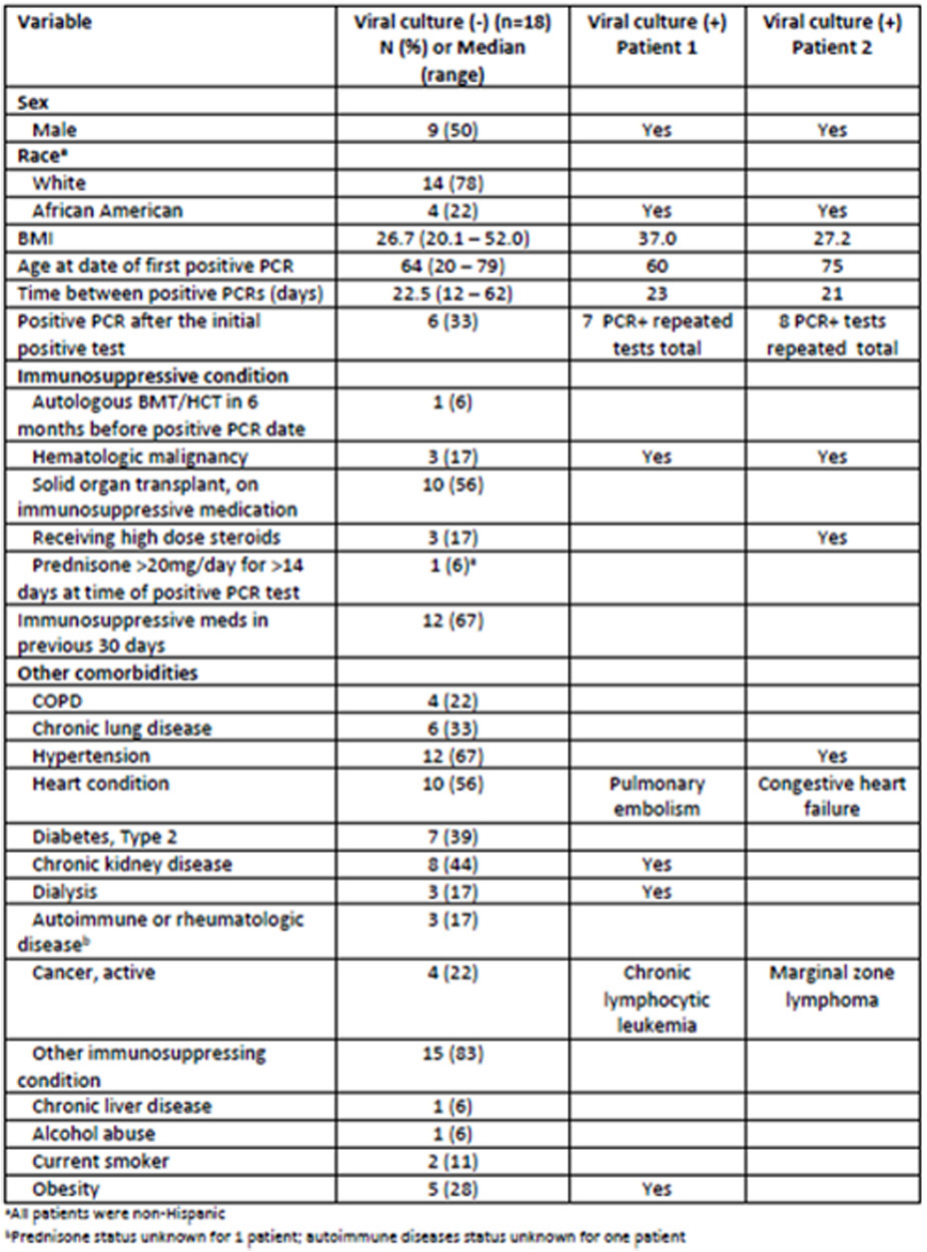

Characteristics of patients with a positive SARS-CoV-2 viral culture

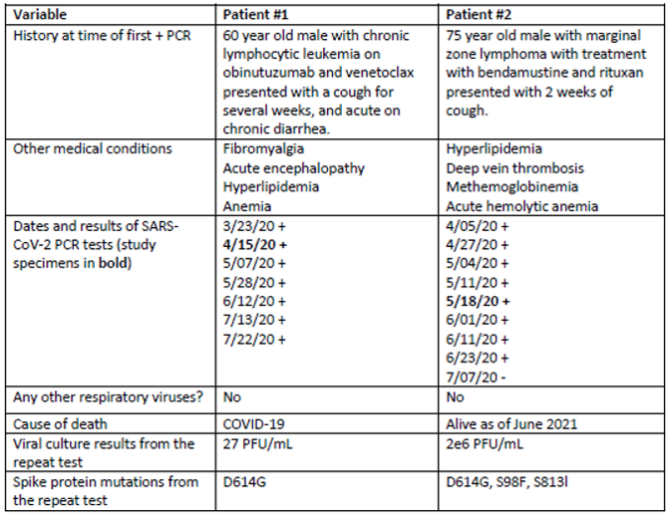

**Conclusion:**

90% of specimens collected > 21 days after an initial pos SARS-CoV-2 PCR did not have viable virus detected on their repeat specimen. The 2 pts with pos viral cultures had active hematologic malignancies treated with an anti-CD20 mAb at the time of COVID-19 diagnosis. One pt had a high concentration of active, viable virus. No known variants of concern were noted in this cohort, collected in Q2 2020, though prolonged replication is a risk for variant development. Further data are needed about risk factors for persistent viable viral shedding & methods to prevent transmission of viable virus from IC hosts.

**Disclosures:**

**Victoria J. Fraser, MD**, **CDC Epicenters** (Grant/Research Support)**Cigna/Express Scripts** (Other Financial or Material Support, Spouse is Chief Clinical Officer)**Doris Duke Fund to Retain Clinical Scientists** (Grant/Research Support, Research Grant or Support)**Foundation for Barnes-Jewish Hospital** (Grant/Research Support, Research Grant or Support)**NIH** (Grant/Research Support, Research Grant or Support) **Victoria J. Fraser, MD**, Centers for Disease Control and Prevention (Individual(s) Involved: Self): Grant/Research Support, Research Grant or Support; Cigna/Express Scripts (Individual(s) Involved: Spouse/Partner): Employee; Doris Duke Charitable Foundation (Individual(s) Involved: Self): Grant/Research Support, Research Grant or Support; National Institutes of Health (Individual(s) Involved: Self): Grant/Research Support, Research Grant or Support; The Foundation for Barnes-Jewish Hospital (Individual(s) Involved: Self): Grant/Research Support, Research Grant or Support **Michael S. Diamond, MD, PhD**, **Carnival Corporation** (Consultant)**Emergent BioSolutions** (Grant/Research Support)**Fortress Biotech** (Consultant)**Immunome** (Advisor or Review Panel member)**Inbios** (Consultant)**Moderna** (Grant/Research Support, Advisor or Review Panel member)**Vir Biotechnology** (Consultant, Grant/Research Support) **Carey-Ann Burnham, PhD**, **BioFire** (Grant/Research Support, Other Financial or Material Support)**bioMerieux** (Grant/Research Support)**Cepheid** (Consultant, Grant/Research Support)**Luminex** (Grant/Research Support)**Roche** (Other Financial or Material Support) **Carey-Ann Burnham, PhD**, BioFire (Individual(s) Involved: Self): Grant/Research Support; bioMerieux (Individual(s) Involved: Self): Grant/Research Support, Scientific Research Study Investigator, Speakers’ bureau; Cepheid (Individual(s) Involved: Self): Consultant, Grant/Research Support, Scientific Research Study Investigator; Luminex (Individual(s) Involved: Self): Scientific Research Study Investigator **Hilary Babcock, MD, MPH, FIDSA, FSHEA**, Nothing to disclose

